# Needle-based storage-phosphor detector radiography is superior to a conventional powder-based storage phosphor detector and a high-resolution screen-film system in small patients (budgerigars and mice)

**DOI:** 10.1038/s41598-019-46546-5

**Published:** 2019-07-11

**Authors:** Wiebke Tebrün, Eberhard Ludewig, Claudia Köhler, Julia Böhme, Michael Pees

**Affiliations:** 1 University Teaching Hospital, Department for Birds and Reptiles, University of Leipzig, An den Tierkliniken 17, 04103 Leipzig, Germany; 2 Department for Companion Animals and Horses, University Clinic for Small Animals, Clinical Unit of Diagnostic Imaging, University of Veterinary Medicine Vienna (Vetmeduni Vienna), Veterinärplatz 1, 1210 Vienna, Austria; 3 University Teaching Hospital, Department for Small Animals, University of Leipzig, An den Tierkliniken 23, 04103 Leipzig, Germany

**Keywords:** Anatomy, Medical research

## Abstract

This method comparison study used radiographs of 20 mice and 20 budgerigars to investigate comparability between computed radiography (CR) and high-resolution screen-film systems and study the effects of reduced radiation doses on image quality of digital radiographs of small patients. Exposure settings used with the mammography screen-film system (SF) were taken as baseline settings. A powder-based storage-phosphor system (CR_P_) and a needle-based storage-phosphor system (CR_N_) were used with the same settings (D/100%) and half the detector dose (D/50%). Using a scoring system four reviewers assessed five criteria per species covering soft tissue and bone structures. Results were evaluated for differences between reviewers (interobserver variability), systems and settings (intersystem variability, using visual grading characteristic analysis). Correlations were significant (p ≤ 0.05) for interobserver variability in 86.7% of the cases. Correlation coefficients ranged from 0.206 to 0.772. For mice and budgerigars, the CR_N_ system was rated as superior to the SF and CR_P_ system for most criteria, being significant in two cases each. Comparing the SF and CR_P_ system, the conventional method scored higher for all criteria, in one case significantly. For both species and both digital systems, dose reduction to 50% resulted in significantly worse scores for most criteria. In summary, the needle-based storage-phosphor technique proved to be superior compared to the conventional storage-phosphor and mammography screen-film system. Needle-based detector systems are suitable substitutes for high-resolution screen–film systems when performing diagnostic imaging of small patients. Dose reduction to 50% of the corresponding dose needed in high-resolution film-screen systems cannot be recommended.

## Introduction

Over the last two decades, the use of digital radiography in veterinary medicine has increased. It offers several advantages over screen-film systems, ranging from electronical storage and image distribution to increased dose efficiency and a greater dynamic range of detector systems^1^. In comparison to screen-film radiography however, digital radiography is limited in its spatial resolution^2^. Image quality in digital radiographs depends on the intrinsic sharpness and noise level of the detector system, with noise being the limiting factor in object detection, while screen-film systems are contrast limited^3^. In digital radiography two different approaches have been developed. On the one hand, there are computed radiography (CR) systems with a storage plate and separate read out process. Then there are direct digital radiography systems, where x-ray photons are directly converted into electrical charges^4^. One established type of CR detector is the conventional powder-based storage phosphor detector (PIP), which consists of small phosphor particles dispersed in a binding agent^5^. A more recently developed CR detector is the needle-based storage phosphor detector (NIP). Here, the phosphor particles form a crystalline needle-structure that is oriented perpendicular to the detector surface^5^. Comparing the technical aspects, NIPs have a higher conversion efficiency than PIPs, resulting in a higher signal-noise-ratio while using identical exposure settings^6^. Smans *et al*.^7^, using a computer model, showed that the threshold-contrast detectability of their simulated NIP system was superior to the also simulated PIP system. In preclinical trials, NIPs and PIPs have been used on phantoms, where NIPs depicted lower contrast levels better than PIPs^8^. When tested on phantoms for chest radiology, NIPs were significantly superior to PIPs regarding image quality and the potential for dose reduction^9,10^. In one phantom study, dose reduction of up to 68% of the initial dose was possible^11^. In clinical trials, a dose reduction of 50% on NIP systems produced images that showed no significant differences in image quality compared to PIP images at 100% of the dose^12^. In neonatal chest radiology a NIP system was preferred by reviewers in comparison to a PIP system, here dose reduction of 20% was possible without detectable loss of image quality^13^.

In veterinary medicine, various digital detector systems have been tested for dogs, cats and large animals such as horses^14–16^. Data concerning the use of digital detector systems for birds, snakes and lizards, with body masses ranging from 123 to 847 g has also been published^17–19^. In general practice, veterinarians are consistently confronted with even smaller patients. Animals like budgerigars and mice, with body masses ranging from 30 to 50 g, make high demands on x-ray technique due to their delicately structured anatomy and their high respiratory rate which demands a shorter exposure time.

To the authors’ knowledge, no studies have been conducted to evaluate the use of computed radiography in patients with body masses lower than 100 g. We wanted to explore the implementation of these methods, since radiography represents an affordable and reliable diagnostic means in standard veterinary practice. The objective of this method comparison study was to investigate whether or not image information generated with CR systems is at least equivalent to that acquired by high-resolution screen-film systems. Furthermore, we wanted to study the effects of a reduced radiation dose in computed radiography on the visibility of structures in these very small animals. As model species, budgerigars and mice were used as representatives for small rodent and pet bird species regularly seen in small animal and specialized practices. Additionally, mice are a commonly used species in laboratory animal science. We especially wanted to include an avian species in our study to take into account some species specific features of avian anatomy, such as air sacks, pneumatized bones and typical position of the intracoelomic organs.

## Materials and Methods

In this study, two different CR storage systems, namely AGFA DX-S (Agfa Healthcare, Bonn, Germany), a needle-based detector technology (referred to as system “CR_N_” for needle-based CR detector), and the powder-based storage phosphor detector system Fuji HR/Philips AC 500 (Philips Healthcare, Hamburg, Germany; system “CR_P_” for powder-based CR detector) were used in comparison to the high-resolution mammography film-screen system KODAK MIN-R S Film (Eastman Kodak Company, Rochester, United States) (referred to as System “SF” for screen-film system). For information on technical equipment and exposure settings see Table 1.

**Table 1:** Technical equipment and exposure settings used in the experimental setup.

System	X-ray system	Detector system	Exposure settings
SF	PHILIPS Bucky Diagnost TH	KODAK MIN-R S Film (Film size: 18 × 24 cm^2^)	40 kVp, 6.3 mAs (31.5 ms) DAP: 0.4 cGy × cm^2^
CR_P_ - 100%	Grid: no Focus-Detector	FUJI HR/PHILIPS AC 500 (Screen size: 18 × 24 cm^2^)	40 kVp, 6.3 mAs (31.5 ms) DAP: 0.4 cGy × cm^2^
CR_P_ 50%	Distance 110 cm		40 kVp, 3.2 mAs (15.8 ms) DAP: 0.2 cGy × cm^2^
CR_N_ - 100%	Focus size: 0.6 × 0.6 mm^2^	AGFA DX-S (Screen size: 18 × 24 cm^2^)	40 kVp, 6.3 mAs (31.5 ms) DAP: 0.4 cGy × cm^2^
CR_N_ - 50%	Filtration: 2.5 mm Al		40 kVp, 3.2 mAs (15.8 ms) DAP: 0.2 cGy × cm^2^

DAP: Dose Area Product.

The radiographs were made using a Bucky-table unit (Philips Bucky Diagnost TH, Philips Healthcare, Hamburg, Germany), but placing the detector on the table and the animals directly on it. Exposure settings were adjusted on the bases of the dose requirements of the film-screen system. The settings identified to generate images of adequate brightness were subsequently used as “100%-dose” in the digital CR systems (D/100%). The halving of the detector dose was achieved by halving the mAs value (D/50%). In the digital systems the displayed values of the dose indicator were used as additional tools for dose control. Dose-Area Product (DAP) measurements were performed for all systems to monitor uniformity of exposure. For the digital CR systems, system-specific processing algorithms were used. In pre-studies, the parameters of these processing algorithms were evaluated with regard to detail visibility. For the Philips Healthcare system (CR_P_) the parameters were set to unsharp mask filtration with gamma type (GT) E, density shift (GS) of 0.27, rotation center (GC) of 1.80, rotation amount (GA) of 1.03, frequency rank (RN) of 9, frequency type (RT) T, frequency enhancement (RE) of 1.00 and Kernel size of 7 with the help of the Workstation Easy Vision Rad Release 4.2. L5 (Philips Healthcare, Hamburg, Germany). For the Afga Healthcare system (CR_N_) the self-adaptive MUSICA 2 software (Agfa Healthcare) and automated algorithms based on default values were used.The settings were consistently used for each detector system, so the process of detection and conversion has to be evaluated as a whole.

### Procedure

In this prospective study, twenty mice with a mean body mass of 38 g, ranging from 32 g to 43 g, and twenty budgerigars with a mean body mass of 41 g, ranging from 33 g to 57 g, were included. The animals did not show any signs of illness regarding their individual history and gross examination that could lead to an expectation of radiologically detectable abnormalities. All radiographs were taken only in right lateral recumbency within the first two minutes of isoflurane induced general anesthesia (isoflurane 2.0–2.5%, 100% oxygen induced via mask). The budgerigars were positioned using a plexiglass avian restraint board. To allow best comparability of the radiographs, all five radiographs of each animal were taken in immediate succession using the same technical settings. The study was approved by the local animal welfare authorities (Landesdirektion Sachsen, No. A01/11) and conducted according to the German animal welfare regulations.

Four reviewers received soft copies of the images taken with two different storage systems and two different doses (100% and 50% each), as well as the radiographs on film. To prevent biased interpretation, observers were unaware of the animal identification, due to the removal of metadata from the digital images and blinding of all images using a DICOM Anonymizer (https://sourceforge.net/projects/dicomanonymizer/) with randomly chosen, unconnected three-digit numbers. The workstation was equipped with two medical gray-scale monitors (EIZO MX240W, matrix: 1920 × 1200 pixel, dot pitch: 0.27 mm; luminance: 320 cd/m^2^, contrast ratio: 850:1; Avnet Technology, Nettetal, Germany). A commercial medical image analyses software was used (GOP-View XR2-T, Contextvision, Stockholm, Sweden).

To be consistent with the practical routine of digital image reading, the radiologists were encouraged to apply the entire workstation functionality to record as much information as possible. Films were analyzed using a light box (Planilux DX, luminance: 4,700 cd/m^2^, Planilux, Warstein, Germany) and a focal spot light (Planilux Irisleuchte 70; luminance: 25,000 cd/m^2^, Planilux, Warstein, Germany) A magnifying glass (×4), and brightness adjustment could be used, if and as necessary. Evaluation time per image was unlimited. The ambient light and other conditions of the viewing environment fulfilled the requirements for medical image interpretation^20^.

### Scoring system

To determine differences between the systems, visual grading characteristics (VGC) analysis, a method previously used in comparable studies, was conducted^17–19^. In VGC studies, predetermined image quality criteria are assessed by multiple reviewers, subjectively rated, here in accordance to an absolute visual grading analysis (VGA), and the scores then compared using a method similar to receiver operating characteristics (ROC) analysis^21,22^.

Criteria were selected with regard to their lack of obviousness, as an overall good rating would prevent the generation of meaningful results. Some criteria were suitable for evaluation of detail rendition (Mice: trachea and spine, Budgerigar: tracheal rings, spine and humerus), others for evaluation of contrast resolution (Mice: heart, lung, abdomen, Budgerigar: lung, kidney).

A four-step scoring system was implemented for each of the five criteria with 1 being the best 4 being the worst score. The observers were trained for their task using a separate set of images. The images were evaluated independently by four observers with a minimum of two years of experience with digital radiography.

**Figure 1:**
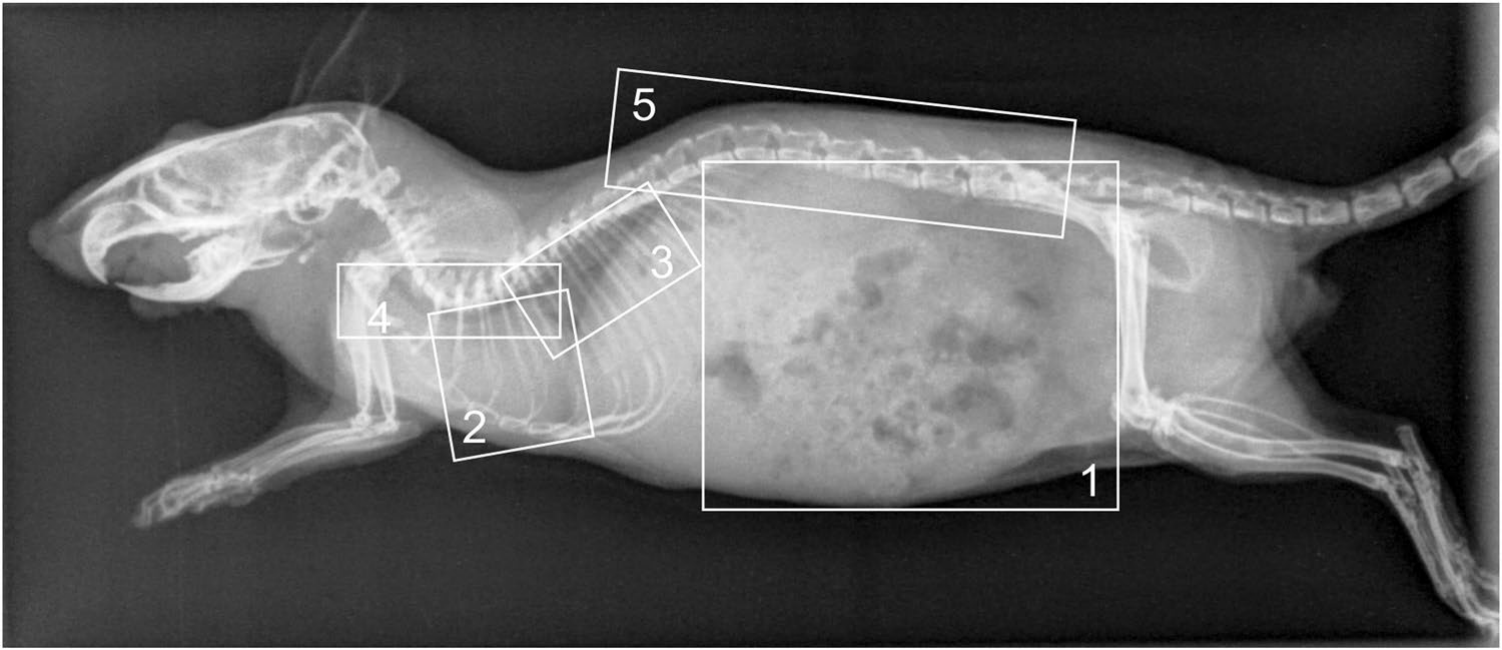
Laterolateral radiographic projection (AGFA DX-S detector, full dose at 40 kVp, 6.3 mAs) of a mouse where the regions for the criteria are defined. Criteria: 1 – Abdomen, 2 – Heart, 3 – Lung, 4 – Trachea, 5 – Spine.

**Figure 2:**
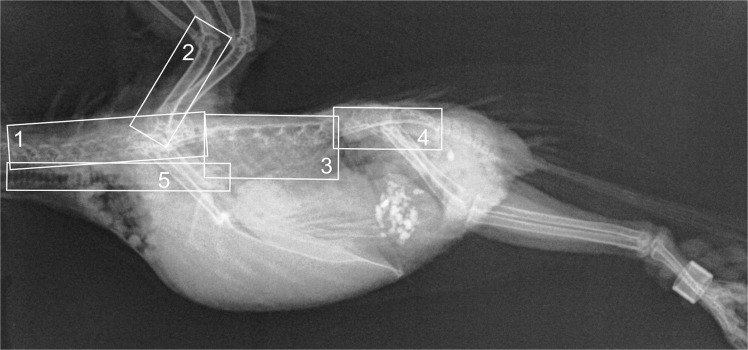
Laterolateral radiographic projection (AGFA DX-S detector, full dose at 40 kVp, 6.3 mAs) of a budgerigar where the regions for the criteria are defined. Criteria: 1 – Cervical spine, 2 – Humerus, 3 – Lung, 4 – Kidney, 5 – Tracheal rings.

For details on the criterion and scoring definition see Tables 2 and 3, and Figs 1 and 2.

**Table 2:** Definition of criteria for radiographic assessment.

Mice	Abdomen	Assessment of the size of abdominal organs (e.g. kidneys)
	Heart	Delineation of caudal heart margin
	Lung	Visualization of physiological lung structure in the caudal pulmonary field
	Trachea	Delineation of trachea and bifurcation to surrounding tissue
	Spine	Visualization of vertebral architecture of the lumbar spine, delineation to surrounding tissue
Budgerigars	Tracheal rings	Delineation of tracheal rings near the cranial thoracic aperture: Visualization and rendition of tracheal rings
	Kidney	Delineation of ventral kidney margin, delineation to surrounding structures, e.g. air sacs, gut walls
	Lung	Visibility of honeycomb structure, Assessment of single “combs”, delineation of honeycomb structure against overlying structures, especially in case of ribs
	Cervical spine	Identification of individual vertebrae, assessment of bone margins, delineation to surrounding tissue
	Humeri	Identification of trabecular and cortical structures, delineation to surrounding structures

**Table 3:** Definition of scores for radiographic assessment.

Score	Assessment
1	optimal impression, structure completely evaluable, no limitation for clinical interpretation
2	good impression, structure evaluable, minor limitation for clinical interpretation
3	acceptable impression, detail representation limited, clinical interpretation restricted
4	insufficient impression, no interpretation possible

### Statistical analysis

In total, 800 assessments of radiographic images were taken into consideration for this study. The Spearman’s rank correlation coefficient was applied (IBM SPSS Statistics 20, IBM, Armonk, NY) for the evaluation of the interobserver variability. Correlation was considered to be significant with p < 0.05 and highly significant with p ≤ 0.001. To evaluate the effect size of correlation, we referred to Cohen^23^. Correlations under 0.1 were considered negligible, correlations between 0.1 and 0.3 were considered low, correlations between 0.3 and 0.5 moderate and correlations over 0.5 were considered high. Additionally, interobserver agreement was calculated using Cohen’s kappa test. Significance levels and effect sizes were considered the same as described above.

Mean values, scoring frequencies and 95%-confidence intervals were calculated to facilitate comparison between systems and reviewers. A receiver operating characteristics (ROC) Analysis was applied for intersystem variability (Sigma Plot 11, Systat Software Inc., San José, CA). The obtained VGC curve graphically demonstrates the comparison of two systems. In case of an equal rating, the curve would be a diagonal resulting in an area under the curve (AUC) of 0.5^18,19^. The more one system is rated superior, the more the curve moves to the respective axis, therefore changing the area under the curve value towards 0.0 or 1.0.

## Results

### Interobserver variability

For criteria in mice, the average scores given by the individual reviewers were 2.82 (reviewer 1), 2.32 (reviewer 2), 3.02 (reviewer 3) and 2.13 (reviewer 4). For criteria in budgerigars, the respective average scores were 2.36, 2.23, 2.90 and 2.28. Interobserver correlations were calculated for all criteria in both species, adding up to 60 rank correlation values, hereafter referred to as cases that were significant in 86.7% (52/60) of the cases, and highly significant in 75.0% (45/60) of the cases. Regarding the evaluation of criteria for mice, the reviewers’ scores correlated in all but three cases significantly with a Spearman’s r ranging from 0.206 to 0.582 and a mean value of 0.354. Those three cases occurred for different criteria and different reviewers. For the budgerigars, the reviewers’ scores correlated for all criteria except for the humerus. For this criterion only one out of six correlations was significant. For the budgerigars, the Spearman’s r ranged from 0.222 to 0.772 with a mean value of 0.503. The interobserver agreement over all criteria was significant for mice and budgerigars between all reviewers. The only exception was an insignificant agreement between reviewer 3 and 4 regarding mice. The agreements ranged from negligible (0.006) to moderate (0.356). When calculated for each criterion individually, significant and low to moderate interobserver agreement could be demonstrated for 46.7% (14/30) of the cases for mice and 50% (15/30) of the cases for budgerigars. For the remainder of the cases the agreement was insignificant.

### Scoring and intersystem variability

#### Mice

Mean scores for the different criteria ranged from 1.80 for the spine through 2.33 for the lung, 2.64 for the heart and 2.68 for the trachea to 3.41 for the abdomen. Mean values for each criterion separated by system/technique are shown in Table 4.

**Table 4:** Summary of results stating the occurrence of scores and mean values.

Species	Structure	System	Occurence of scores					
			1	2	3	4	mean value	95% Confidence interval
Mice	Abdomen	CR_P/100%_	2	8	26	44	3.40	3.23–3.57
		CR_P/50%_	1	8	16	55	3.56	3.40–3.72
		SF	2	12	19	47	3.39	3.20–3.57
		CR_N/100%_	2	16	20	41	3.27	3.07–3.46
		CR_N/50%_	0	13	18	49	3.45	3.28–3.62
	Heart	CR_P/100%_	5	26	40	9	2.66	2.49–2.83
		CR_P/50%_	3	20	36	21	2.94	2.76–3.12
		SF	7	32	36	5	2.49	2.32–2.65
		CR_N/100%_	10	33	30	7	2.43	2.24–2.61
		CR_N/50%_	1	31	42	6	2.66	2.52–2.80
	Lung	CR_P/100%_	7	43	28	2	2.31	2.16–2.46
		CR_P/50%_	2	35	40	3	2.55	2.41–2.69
		SF	1	64	15	0	2.18	2.08–2.27
		CR_N/100%_	7	55	17	1	2.15	2.02–2.28
		CR_N/50%_	0	41	39	0	2.49	2.38–2.60
	Trachea	CR_P/100%_	5	23	40	12	2.74	2.56–2.91
		CR_P/50%_	4	6	41	29	3.19	3.01–3.36
		SF	3	36	39	2	2.50	2.36–2.64
		CR_N/100%_	14	38	27	1	2.19	2.02–2.35
		CR_N/50%_	3	20	48	9	2.79	2.63–2.94
	Spine	CR_P/100%_	29	47	4	0	1.69	1.56–1.81
		CR_P/50%_	23	39	18	0	1.94	1.78–2.10
		SF	40	38	2	0	1.53	1.40–1.65
		CR_N/100%_	25	49	6	0	1.76	1.63–1.89
		CR_N/50%_	19	36	25	0	2.08	1.91–2.24
Budgerigars	Cervical spine	CR_P/100%_	8	32	32	8	2.50	2.32–2.68
		CR_P/50%_	2	13	40	25	3.10	2.93–3.27
		SF	4	31	40	5	2.58	2.42–2.73
		CR_N/100%_	13	33	23	8	2.34	2.14–2.54
		CR_N/50%_	4	15	40	21	2.98	2.79–3.16
	Humerus	CR_P/100%_	27	41	12	0	1.81	1.66–1.96
		CR_P/50%_	3	59	18	0	2.19	2.08–2.29
		SF	19	51	10	0	1.89	1.76–2.02
		CR_N/100%_	17	53	7	0	1.87	1.75–1.99
		CR_N/50%_	6	51	23	0	2.21	2.09–2.34
	Lung	CR_P/100%_	3	29	41	7	2.65	2.50–2.80
		CR_P/50%_	0	4	51	25	3.26	3.14–3.38
		SF	5	36	34	5	2.49	2.33–2.65
		CR_N/100%_	12	37	28	0	2.21	2.05–2.37
		CR_N/50%_	0	16	51	13	2.96	2.83–3.10
	Kidney	CR_P/100%_	15	28	26	11	2.41	2.20–2.62
		CR_P/50%_	4	25	31	20	2.84	2.65–3.03
		SF	17	31	22	10	2.31	2.10–2.52
		CR_N/100%_	18	30	18	11	2.29	2.06–2.51
		CR_N/50%_	10	27	31	12	2.56	2.36–2.76
	Tracheal rings	CR_P/100%_	21	27	28	4	2.19	1.99–2.38
		CR_P/50%_	3	17	38	22	2.99	2.81–3.17
		SF	22	33	21	4	2.09	1.90–2.28
		CR_N/100%_	32	27	18	0	1.82	1.64–2.00
		CR_N/50%_	11	24	38	7	2.51	2.33–2.70

At 100% of the dose, the CR_N_ system received a mean score of 2.36, the CR_P_ system a score of 2.56 and the mammography film system SF a score of 2.42. The CR_N_ system was evaluated as superior to the SF system for four out of five criteria, with the score differing significantly in one case (p = 0.014). Regarding the one criterion in which the SF system scored better, this was also significant (p = 0.02). Also, system CR_N_ scored higher than the CR_P_ system for four out of five criteria, one of which was highly significant (p ≤ 0.001). When comparing the SF system with the CR_P_ system, the conventional method scored higher for all five criteria with the differences being significant for one criterion (p = 0.048).

At 50% of the dose, the CR_N_ system received a mean score of 2.69, the CR_P_ system a score of 2.84. When comparing the CR_N_ system at 100% and 50%, the 100% dose always received a better score, being significantly better for three out of five criteria (p = 0.011, p ≤ 0.001, p ≤ 0.001). When comparing the CR_P_ system at 100% and 50%, the 100% dose always received better scores. Here, the differences were significant for four out of five criteria (p = 0.001 for one and p = 0.04 for the other three).

#### Budgerigars

Mean scores for the different criteria ranged from 1.99 for the humerus through 2.32 for the tracheal rings, 2.48 for the kidney and 2.70 for the cervical spine to 2.72 for the lung. Mean values separated by criterion and system/technique can be found in Table 4.

At 100% of the dose, the CR_N_ system received a mean score of 2.11, the CR_P_ system a score of 2.31 and the SF system a score of 2.27. The CR_N_ system was evaluated as superior to the mammography screen-film system SF for five out of five criteria, with the score differing significantly in one case (p = 0.024). Also, the CR_N_ system scored higher than the CR_P_ system for four out of five criteria, one of which was highly significant (p ≤ 0.001). When comparing the SF system with the CR_P_ system, the conventional method scored higher for three criteria, the CR_P_ system for 2 criteria. The differences were not significant.

At 50% of the dose, the CR_N_ system received a mean score of 2.64, the CR_P_ system a score of 2.88. When comparing the CR_N_ system at 100% and 50%, the 100% dose always received a better score, the difference being highly significant for four out of five criteria (p ≤ 0.001, p = 0.002, p ≤ 0.001, p = 0.002). When comparing the CR_P_ system at 100% and 50%, the 100% dose always received better scores, as well. Here the differences were significant to highly significant for five out of five criteria (p = 0.01, p ≤ 0.001, p ≤ 0.001, p = 0.04, p = 0.03).

All in all, the systems scored higher at full dose than at half dose, for both mice and budgerigars. Further details are shown in Table 5.

**Table 5:** Summary of the statistical analyses stating significant occurrences in intersystem variability through statistically calculated AUC values.

Species	Criteria	System									
		CR_P/100%_ - CR_N/100%_	CR_P/100%_ - SF	SF - CR_N/100%_	SF - CR_N/50%_	CR_P/50%_ - SF	CR_P/50%_ - CR_N/50%_	CR_P/100%_ - CR_P/50%_	CR_N/100%_ - CR_N/50%_	CR_P/100%_ - CR_N/50%_	CR_P/50%_ - CR_N/100%_
Mice	Abdomen	0.46	0.51	0.46	0.51	0.45	0.46	0.56	0.56	0.52	0.40^*^
	Heart	0.42	0.44	0.48	0.56	0.35^**^	0.40^*^	0.59^*^	0.58	0.49	0.34^**^
	Lung	0.43	0.44	0.49	0.65^**^	0.33^**^	0.47	0.59^*^	0.65^**^	0.57	0.33^**^
	Trachea	0.32^**^	0.41^*^	0.39^*^	0.62^*^	0.25^**^	0.34^**^	0.66^**^	0.71^**^	0.52	0.18^**^
	Spine	0.53	0.43	0.60^*^	0.70^**^	0.35^**^	0.55	0.59^*^	0.62^*^	0.64^**^	0.44
Budgerigars	Cervical spine	0.44	0.53	0.41	0.65^**^	0.40^*^	0.54	0.62^*^	0.70^**^	0.66^**^	0.34^**^
	Humerus	0.53	0.53	0.49	0.63^**^	0.37^**^	0.52	0.65^**^	0.64^**^	0.66^**^	0.36^**^
	Lung	0.35^**^	0.44	0.40^*^	0.68^**^	0.22^**^	0.38^**^	0.73^**^	0.76^**^	0.62^*^	0.15^**^
	Kidney	0.43	0.43	0.49	0.58	0.35^**^	0.42	0.59^*^	0.59	0.51	0.34^**^
	Tracheal rings	0.43	0.43	0.50	0.64^**^	0.34^**^	0.48	0.60^*^	0.64^**^	0.57	0.34^**^

*Significant (p ≤ 0.05), **highly significant (p ≤ 0.001).

Interpretation: an equal assessment of the criterion would result in a value of 0.5. The more one system is superior, the more the value tends to 1.0 (first system) or 0.0 (second system). “CR_N_” refers to AGFA DX-S (Agfa Healthcare, Bonn, Germany) standing for digital needle-based detector, “CR_P_” refers to Fuji HR / Philips AC 500 (Philips, Hamburg, Germany) standing for digital detector and “SF” refers to KODAK MIN-R S (Kodak, Stuttgart, Germany) for a conventional mammography screen-film. The numbers 100% and 50% refer to the percentage of dosage used in the trial. Significance levels refer to the deviation from the AUC value 0.5 when applying the ROC-analysis.

## Discussion

### Evaluation methods

Visual grading characteristic (VGC) analysis is a method to evaluate the performance of different radiographic systems and has previously been applied in similar studies^17–19^. Evaluating physical parameters is important to standardize examination procedures but it does not necessarily allow predictions about the clinical performance of radiographs^24^. Visual grading in contrast uses anatomical criteria for a visibility assessment^25^, therefore offering an objective link to clinical interpretations. Although we used a standardized study setup, there is still potential bias arising from individual differences, in this case of the reviewers rating the images^26^. The reviewers were chosen with regard to their experiences in radiology, as well as experience in the interpretation of avian radiographs. A training session was held beforehand to reduce divergence in scoring. However, the mean scores varied by about one point for mice and about half a point for budgerigars leading to a low to moderate agreement. While the reviewers were not necessarily expected to give the same scores, which is reflected in the level of agreement, it was still interesting whether the tendencies of scoring were the same. Therefore, even though one reviewer marked the radiographs significantly worse than the other three, correlations were significant for all criteria, except for the criterion humerus in budgerigars. This criterion always received good scores regardless of the system or dose, displaying a limited discriminatory power when trying to draw comparisons between different settings. Therefore it can be assumed that the criterion did not fully meet the expectations for VGC analysis. In contrast, the budgerigars’ cervical spine, which consists of more delicately structured bones, scored lower. Here the high number and small size of the single vertebral bodies could influence interpretability. The other criteria in budgerigars ranged between these extremes. Due to the air sacs, radiographic images have good coelomic contrast and superimpositions in the coelomic cavity are less extensive than in the mammalian abdomen, possibly explaining why the mean scores for mice differed more than the mean scores for budgerigars. Here, the criterion spine received an exceptionally good score, while the criterion abdomen scored lowest. A probable explanation is that discerning different soft tissue organs in the abdomen is more challenging. In total, the choice of criteria enabled the provision of a broad distribution of scores.

### Comparison of radiography systems

The systems tested in this study are of great relevance for the use in veterinary practice, as they represent both a conventional technique that has long been recommended for small patients as well as an established computed radiography system. The needle-based storage phosphor system is a promising technique that is commonly used in human medicine, but is not yet common in veterinary practice. Relating to the mean scores at 100% dosage, the CR_N_ system ranked first, the mammography SFsystem second and the CR_P_ system third. Concerning the powder- (CR_P_) and needle-based (CR_N_) storage phosphor detectors, this corresponds with the findings of studies on human chest radiography^8,9,11,12^. Due to the production process, powder-based storage phosphor detectors like the CR_P_ show a weakness concerning image sharpness, as light scatters on the powder particles. In the mice radiographs, for example, the CR_P_ system was evaluated significantly worse than the other two systems regarding the criterion trachea. The trachea is a small-sized structure which is often overlaid by surrounding tissue. This demonstrates the limitations of this specific digital storage system.

When it comes to needle-based storage phosphor detectors, the needles work like optical fibers, allowing higher image sharpness due to less scattering of photons during exposure and improved conduction of photons to the detector resulting in little extinction on the way^5^. As mentioned above the criterion trachea in the mice radiographs, for example, scored significantly higher for the CR_N_ system than for both the SF and CR_P_ system, leading to the conclusion that needle-based detectors have a higher potential to depict small-sized soft tissue structures. Regarding the budgerigar radiographs, significant differences occurred for the criterion lung, where the CR_N_ system scored better than the SF system and the CR_P_ system, indicating that delicate soft tissue structures like the honeycomb structure of the lung are also best depicted with needle-based detectors.

The mammography film system ranked in the middle. This shows parallels to an early study of Bacher et al.^27^, where the mammography system also managed to score better than a powder-based storage phosphor detector. For instance, the criterion spine in the mice radiographs proved to be evaluated best on the conventional mammography system. Even though mammography films are optimized for discerning soft tissues, their spatial resolution is superior in comparison to digital radiographic systems, so they have an advantage in depicting miniscule bone structures. However, studies have shown that detail detection is sometimes scored better using the digital system^28^. Due to their superior dynamic range, the digital systems score better in terms of soft tissue structures. Similar experiences have been made in preceding studies^17^.

When talking about digital radiography, direct digital radiography such as flat panel detectors, in contrast to the computed radiography systems used in this study, also need to be included in the discussion as they are widely used in practice. Studies have shown, that mammography films performed worse than flat panel detector system^27^. However,when comparing NIPs and the more modern flat panel detectors, reports vary. Tests on human cadavers suggested that flat panel detector performance and dose reduction potential is inferior when compared to NIPs^4^. Another source stated that calcification detection is significantly reduced when using NIPs in comparison to a flat panel system^29^.

### Effects of dose reduction

Dose reduction was predominantly compared within each system. Data showed that in both species and both systems dose reduction lead to significantly worse scores for the majority of the criteria. Exceptions from the image deterioration were the criteria kidney for budgerigars and heart for mice using the CR_N_ system and the criterion abdomen for mice in both systems. While the kidneys, which are surrounded by air sacs, seemed to be contrasted enough to always score in the same range regardless of the radiation dose, the criterion abdomen generally scored poorly because of a lack of contrast between the abdominal organs.

In comparison to the SF system at 100% of the dose, dose reduction within the CR_N_, as well as the CR_P_ system lead to significantly lower scores for nearly all the criteria. We based the exposure settings on the dose requirements of the film-screen system and analogically used the same setting for the digital systems. Mammography films traditionally require higher doses due to their sensitivity class and higher resolution than standard film-screens^30^, so it is surprising that the same high doses are needed for digital imaging in small patients to receive similar scores in the assessment.

Based on these results, dose reduction cannot be recommended for digital radiographic imaging in small patients. However, reduction was assessed in only two steps (100%, 50%), which gives only a rough impression of the effect of dose reduction. Further studies that focus on the gradual effect of dose reduction on image quality are needed. A transition from conventional to digital radiographic imaging does not automatically entail dose reduction for small patients.

Other studies considered dose reduction possible for animals with a body mass of around 400 to 500 g^17,18^, while dose reduction in animals with 200 g body mass already led to detectable loss of quality^19^. An adequate radiation dose, implementing the ALARA principle, is therefore especially essential in very small animals to achieve radiographic images that allow for sufficient clinical evaluation.

### Limitations of the study

The study results were very similar for two species from very diverging classes, indicating that the conclusions might also be relevant for other small species, even belonging to other classes, such as reptiles. Although we used a limited number of patients (n = 20) and only two species were assessed separately, the obtained results provide valid recommendations for practitioners in small mammal and avian medicine.

## Conclusion

The digital needle-based storage phosphor technique (CR_N_) proved to be superior in depiction of different delicate structures of small animals with a mean body weight of 30 to 50 g, in comparison with the conventional storage-phosphor system (CR_P_) and – except for the detection of delicate bone structures – also in comparison to the high-resolution screen-film system (SF). Needle-based detector systems can therefore be recommended as a substitute for conventional systems when performing diagnostic imaging in very small patients. As in conventional radiography, an appropriate radiation dose is also essential within the digital systems to achieve images that allow optimal interpretation.
